# A novel Hsp90 inhibitor AT13387 induces senescence in EBV-positive nasopharyngeal carcinoma cells and suppresses tumor formation

**DOI:** 10.1186/1476-4598-12-128

**Published:** 2013-10-24

**Authors:** King Chi Chan, Choi Man Ting, Pui Shan Chan, Ming Chu Lo, Kwok Wai Lo, Jayne E Curry, Tomoko Smyth, Anne Wing Mui Lee, Wai Tong Ng, George Sai Wah Tsao, Ricky Ngok Shun Wong, Maria Li Lung, Nai Ki Mak

**Affiliations:** 1 Department of Biology, Hong Kong Baptist University, Hong Kong, P.R. China; 2 Department of Anatomical and Cellular Pathology, State Key Laboratory in Oncology in South China, The Chinese University of Hong Kong, Hong Kong, P.R. China; 3 Astex Pharmaceuticals, Cambridge, UK; 4 Clinical Oncology, Pamela Youde Nethersole Eastern Hospital, Hong Kong, P.R. China; 5 Department of Anatomy, University of Hong Kong, Hong Kong, P.R. China; 6 Department of Clinical Oncology, University of Hong Kong, Hong Kong, P.R. China; 7 Center for Nasopharyngeal Carcinoma Research, University of Hong Kong, Hong Kong, P.R. China

**Keywords:** AT13387, Hsp90 inhibitor, Senescence, Antitumor, Nasopharyngeal carcinoma

## Abstract

**Background:**

Nasopharyngeal carcinoma (NPC) is an epithelial malignancy strongly associated with Epstein-Barr virus (EBV). AT13387 is a novel heat shock protein 90 (Hsp90) inhibitor, which inhibits the chaperone function of Hsp90 and reduces expression of Hsp90-dependent client oncoproteins. This study aimed to evaluate both the *in vitro* and *in vivo* antitumor effects of AT13387 in the EBV-positive NPC cell line C666-1.

**Results:**

Our results showed that AT13387 inhibited C666-1 cell growth and induced cellular senescence with the downregulation of multiple Hsp90 client oncoproteins EGFR, AKT, CDK4, and restored the protein expression of negative cell cycle regulator p27. We also studied the ability of AT13387 to restore p27 expression by downregulation of AKT and the p27 ubiquitin mediator, Skp2, using AKT inhibitor and Skp2 siRNA. In the functional study, AT13387 inhibited cell migration with downregulation of a cell migration regulator, HDAC6, and increased the acetylation and stabilization of α-tubulin. We also examined the effect of AT13387 on putative cancer stem cells (CSC) by 3-D tumor sphere formation assay. AT13387 effectively reduced both the number and size of C666-1 tumor spheres with decreased expression of NPC CSC-like markers CD44 and SOX2. In the *in vivo* study, AT13387 significantly suppressed tumor formation in C666-1 NPC xenografts.

**Conclusion:**

AT13387 suppressed cell growth, cell migration, tumor sphere formation and induced cellular senescence on EBV-positive NPC cell line C666-1. Also, the antitumor effect of AT13387 was demonstrated in an *in vivo* model. This study provided experimental evidence for the preclinical value of using AT13387 as an effective antitumor agent in treatment of NPC.

## Background

Nasopharyngeal carcinoma (NPC) is a malignancy arising from the epithelial cells of the nasopharynx. It has a distinct geographic distribution with a remarkably high disease incidence in southern China and Southeast Asia with more than 50,000 new cases each year [1]. Apparently, all NPC is associated with the Epstein-Barr virus (EBV) latent infection, indicating the role of EBV in NPC pathogenesis [2]. However, most of the NPC cell lines had lost the EBV genome after a long time *in vitro* passage. C666-1 is the NPC cell line consistently maintaining the native EBV genome and referred as a suitable model for studies of EBV-associated NPC [3]. Nowadays, combined radiotherapy and chemotherapy are used for the treatment of NPC patients [4,5]. Most contemporary series reported very encouraging results with locoregional control exceeding 90%, but distant failure remains high and more potent systemic therapy is needed.

Heat shock protein 90 (Hsp90) is a molecular chaperone involved in the maturation and stabilization of over 200 oncogenic client proteins crucial for oncogenesis [6-8]. Hsp90 inhibitors exert the antitumor effect by blocking the ATP binding domain of Hsp90 to abolish the Hsp90 chaperone function and leading to proteasomal degradation of the oncogenic client proteins. In tumor cells, the dependency of oncoproteins on the chaperone function of Hsp90 is much higher than in normal cells, and the binding affinity of Hsp90 inhibitor to Hsp90 was 100-fold higher in tumor cells than in normal cells [9-11]. For this reason, inhibition of the Hsp90 machinery is considered as a potent strategy in cancer therapies [12].

AT13387 is a small-molecule inhibitor of Hsp90 developed by Astex Pharmaceuticals Inc through fragment-based drug screening against the ATP-binding domain of Hsp90 [13]. Several studies also reported AT13387 as an effective antitumor agent in both the *in vitro* and *in vivo* cancer models, such as gastrointestinal stromal tumor (GIST) and non-small cell lung cancer (NSCLC) [14,15]. AT13387 clinical activity against GIST was demonstrated in the Phase I and Phase II trials (ClinicalTrials.gov Identifier: NCT00878423 [16] and NCT01294202 [17], respectively), and further clinical trials in prostate (NCT01685268) and lung cancer (NCT01712217) in combination with standard of care are ongoing.

In NPC, many of the aberrantly overexpressed oncoproteins such as EGFR, AKT, and CDK4 are known Hsp90 client proteins [12,18,19]. We hypothesize that targeting the chaperone function of Hsp90 in NPC cells can lead to downregulation of multiple crucial oncoproteins and regression of tumor. Therefore, we aim to study the tumor suppressive efficacy of AT13387 in the C666-1 EBV-positive NPC cell line and provide preclinical evidence of using AT13387 as a novel antitumor agent in treatment of NPC.

## Results

### Growth inhibitory effect of AT13387 on the EBV-positive NPC cell line C666-1

**Figure 1:**
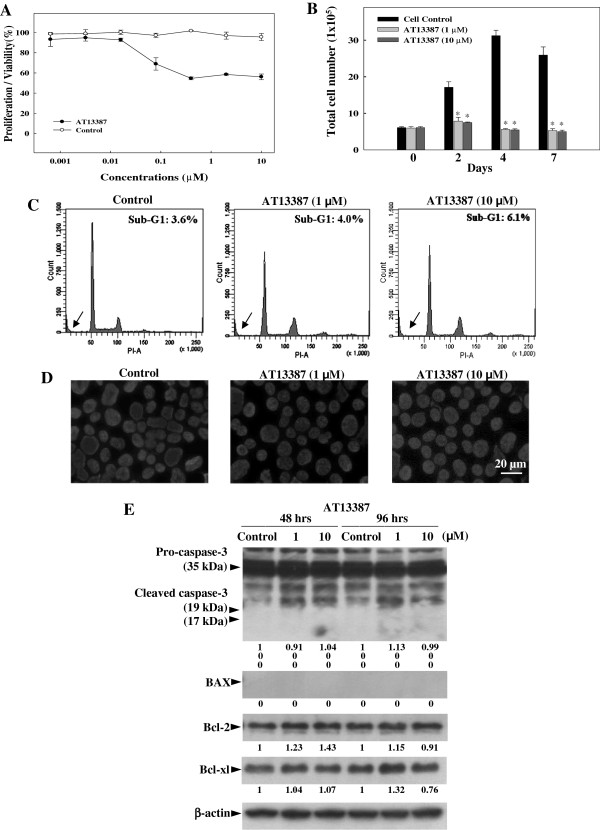
**Effect of AT13387 on EBV-positive NPC cell line C666-1. (A)** MTT assay showing dose dependent inhibition of C666-1 cells growth after 48 hrs of AT13387 treatment. **(B)** Cell growth assay showing the kinetics of growth inhibition determined by counting number of viable C666-1 cells on days 2, 4 and 7 after AT13387 treatment. Results are expressed as the mean ± S.D. of three separate trials; *p < 0.05. **(C)** DNA content analysis and **(D)** DAPI nuclei staining showed no significant apoptotic phenotype in AT13387-treated C666-1 after 48 hrs. Arrow indicated the sub-G1 peak of DNA profile. Scale bar = 20 μm. **(E)** Western-blotting analysis showing no significant change of pro-apoptotic and anti-apoptotic proteins in AT13387-treated C666-1 after 48 hrs and 96 hrs. Value represented the arbitrary unit of band intensity after normalization with β-actin.

The growth inhibitory effect of AT13387 on the EBV-positive NPC cell line C666-1 was demonstrated in the MTT assay (Figure 1A) and cell growth assay (Figure 1B). In MTT assay, C666-1 was treated with various concentrations of AT13387 for 48 hours. Results showed that AT13387 inhibited the growth of C666-1 dose-dependently when compared with untreated control. Maximum inhibition of cell growth was observed in C666-1 treated with 1 μM to 10 μM AT13387. Therefore, 1 μM and 10 μM AT13387 were chosen for further analysis. In the cell growth assay, number of viable C666-1 cells after 1 μM and 10 μM AT13387 treatment for 2 to 7 days were determined by cell counting. The total number of AT13387-treated C666-1 cells at day-2, 4, and 7 was similar to the initial number of C666-1 cells at day 0, showing no growth of AT13387-treated C666-1 cells, while the control cells continued to grow till Day 4 after which it reached a plateau. The total number of AT13387-treated C666-1 cells at day-2, 4, and 7 was significantly lower than their respective control groups (*p < 0.05). Next, we tried to determine whether the mode of growth inhibition of AT13387 on C666-1 cells was due to induction of apoptosis. However, DNA content analysis of 1 μM and 10 μM AT13387-treated C666-1 showed no clear increase of sub-G1 peak after 48 hours (Figure 1C) and DAPI nuclei staining of AT13387-treated C666-1 did not reveal the typical appearance of apoptotic cells with chromatin condensation and fragmentation (Figure 1D). Results showed no apparent apoptotic phenotype in the AT13387-treated C666-1 cells. In addition to the nuclear staining and DNA content analysis, the expression of pro-apoptotic proteins (cleaved form of Caspase-3 and BAX) and anti-apoptotic proteins (Bcl-2 and Bcl-xl) were analysed (Figure 1E). The Western blotting result showed after 48 hours and 96 hours of AT13387 treatment, cleaved forms of caspase-3 (19 kDa and 17 kDa) and BAX pro-apoptotic proteins were not expressed in AT13387-treated C666-1. The expression of anti-apoptotic proteins Bcl-2 and Bcl-xl in AT13387-treated C666-1was also not decreased, indicating that induction of apoptosis is not the major mechanism in AT13387-treated C666-1 cells.

### AT13387 induces senescence in C666-1

**Figure 2:**
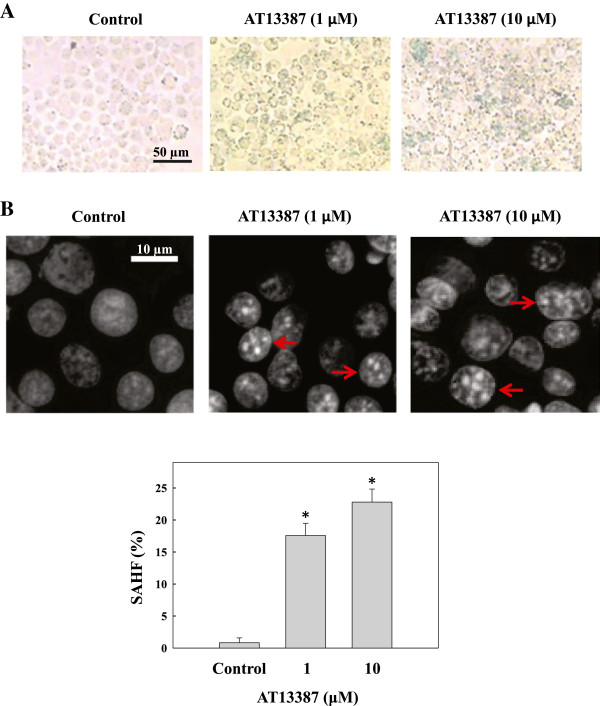
**Characterization of senescence phenotypes in AT13387-treated C666-1. (A)** Staining of senescence-associated β-galactosidase (SA-β-gal). Senescent cells were identified as cells stained blue after 72 hrs of AT13387 treatment. Scale bar = 50 μm. **(B)** DAPI staining and quantification of senescence-associated heterochromatic foci (SAHF). Upper panel: microscopic image showing formation of SAHF in C666-1 cells with AT13387 treatment for 96 hrs. Scale bar = 10 μm, red arrow indicated cells with typical SAHF. Lower panel: bar chart showing the percentage of cells with SAHF. At least 200 cells were counted from different microscopic fields for each treatment. Results were expressed as the mean ± S.D. of three independent experiments; *p < 0.05.

Cellular senescence is a permanent and irreversible process in the induction of cell growth arrest without induction of massive cell death [20,21]. Chemotherapy-induced senescence is one of the tumor suppression mechanisms in antitumor therapy. Since an apoptotic response was not observed in the C666-1 cells in the mentioned AT13387 experiments, we sought to determine whether the growth inhibitory effect of AT13387 was due to the induction of cellular senescence. C666-1 cells treated with AT13387 for 72 hours were then stained for the senescence-associated β-galactosidase (SA-β-gal). Results in Figure 2A showed that SA-β-gal-positive cells stained in blue were observed in cells after AT13387 treatment. Since the blue staining of SA-β-gal is weakly expressed and difficult to quantify, the formation of senescence-associated heterochromatin foci (SAHF) [22], was then performed. Compact punctuate DAPI-stained SAHF were clearly seen and quantified in AT13387-treated C666-1 cells after 96 hours (*p < 0.05) (Figure 2B). Results from this study indicated that AT13387 induced cellular senescence in the C666-1 cells.

### Western-blotting analysis of senescence and growth associated Hsp90 client oncoproteins and the re-expression of p27 after AT13387 treatment

**Figure 3:**
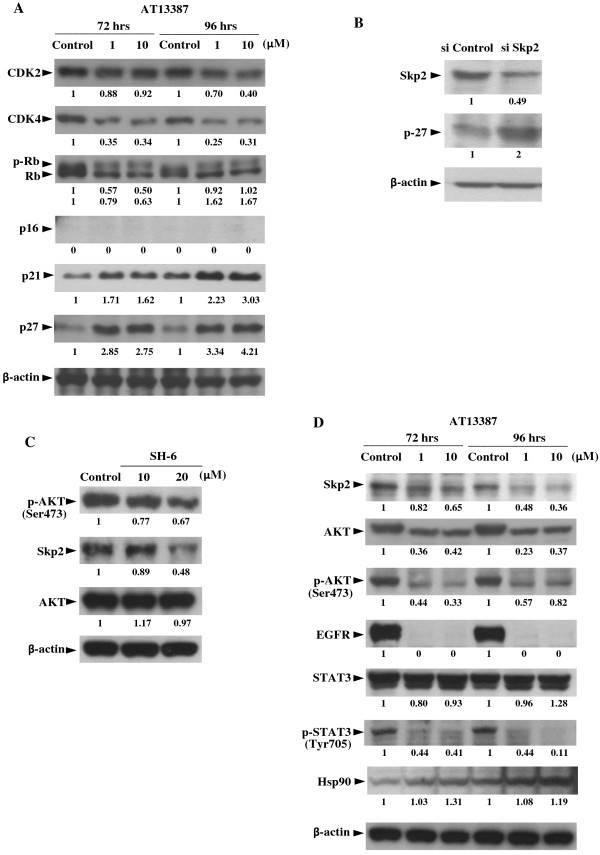
**Western-blotting analysis of Hsp90 client proteins and F-box protein S-phase kinase 2 (Skp2) in AT13387-treated C666-1. (A)** Expression of senescence and cell cycle associated Hsp90 client proteins in C666-1 after 72 hrs and 96 hrs AT13387 treatment. **(B)** Knockdown of Skp2 by siRNA leaded to upregulation of p27 in C666-1. **(C)** Downregulation of p-AKT (Ser473) and Skp2 by AKT inhibitor. **(D)** Downregulation of Skp2 and Hsp90 client oncoproteins AKT, p-AKT (Ser473), EGFR, and p-STAT3 after AT13387 treatment. Value represented the arbitrary unit of band intensity after normalization with β-actin.

Induction of cellular senescence is usually associated with the altered expression of cell cycle regulators. We first analyzed the expression of senescence and cell cycle associated Hsp90 client proteins CDK2 and CDK4 in AT13387-treated C666-1 cells. At the concentration of 1 μM (72 hours), the expression level of CDK2 and CDK4 was about 88% and 35% of the control value, respectively (Figure 3A). At the concentration of 10 μM, the protein expression level of CDK2 (96 hours) was about 40% of the control group, indicating that AT13387 exerted a greater inhibitor effect on the expression of CDK4 than the CDK2. Rb protein is the downstream target of CDK2 and CDK4, and the state of Rb phosphorylation is known to regulate the cell growth and cellular senescence. Furthermore, the activity of CDK2 and CDK4 is regulated by the cell cycle regulators p16, p21, and p27. We then measured the expression of p16, p21, p27 and the phosphorylated form of Rb protein (p-Rb, inactive form) in AT13387-treated C666-1. Although the upregulated expression of p16 is generally considered as a major effector in the induction of senescence, p16 was not expressed by both untreated and AT13387-treated C666-1 cells. This can be explained by the fact that the CDKN2A-CDKN2B gene cluster on 9p21 encoding p16 is a highly susceptible loci in NPC [23], so that the re-expression of p16 is not observed in the commonly deleted loci in C666-1. Meanwhile, the expression level of important senescence regulators p21 and p27 was increased in cells after AT13387 treatment. At the concentration of 10 μM, there was about a 1.62 and 2.75-fold increase in the expression of p21 and p27, respectively at 72 hours after AT13387 treatment, and the increase in p21 and p27 expression were also accompanied by a decrease in the expression of p-RB. However, the reduction in the level of p-RB was not apparent at 96 hours after the treatment. Taken together, upregulation of p21 and p27 was well correlated with the downregulation of CDK4 in AT13387-treated C666-1 cells.

The negative cell cycle regulator p27 has previously been reported as a commonly downregulated tumor suppressive protein in NPC. In order to further study the mechanism of resotration of p27 protein expression in AT13387-treated C666-1 cells, we first measured the p27 mRNA expression by real-time quantitative PCR. However, the p27 mRNA level was unchanged by 72 hours treatment with AT13387 (data not shown), then we focused on the regulation of p27 at the protein level. The degradation of p27 protein is known to require the interaction between p27 and the F-box protein S-phase kinase 2 (Skp2) in the SCF^skp2^ complex [24]. Since p27 is a normal physiological target of Skp2 for ubiquitination, we then studied the inversed expression of Skp2 and p27 by treating C666-1 cells with Skp2 siRNA. Results in Figure 3B showed that the expression of p27 proteins was increased in the Skp2 siRNA-treated C666-1. It has previously been shown that Skp2 is highly expressed in NPC tumor with poor prognosis [25,26], and the stability of Skp2 is regulated by AKT [27]. We then measured the protein expression of Skp2 after adding the AKT inhibitor SH-6 in C666-1. Results in Figure 3C showed with the downregulation of p-AKT, the Skp2 is coordinately downregulated in SH-6 treated C666-1. We then further determined the expression of Skp2 and AKT in AT13387-treated C666-1 cells. Figure 3D showed that the expression of Skp2, AKT, and phosphorylated form of AKT (p-AKT) were all reduced in the AT13387-treated C666-1 cells. This observation suggested that the ability of AT13387 to restore p27 protein expression may due to downregulation of p27 ubiquitination mediator Skp2 through downregulating AKT and p-AKT.

Apart from AKT, EGFR is one of the most commonly overexpressed oncoproteins in NPC [18,19]. Targeting EGFR has been suggested as a new therapeutic treatment in NPC and EGFR is also a known Hsp90 client oncoprotein. In this study, AT13387 significantly reduced EGFR and its downstream target p-STAT3 in C666-1 (Figure 3D). It is worthy to note that AT13387 is designed to block the Hsp90 chaperon function, therefore the expression level of Hsp90 was not affected by AT13387. Taken together with the downregulation of CDK4, AKT, and Skp2, AT13387 can deplete multiple oncoproteins and restore the tumor suppressive protein p27 in EBV-positive NPC cell line. This result supported the potential use of AT13387 as an antitumor agent in NPC by simultaneously targeting multiple NPC oncoproteins.

### Inhibition of tumor cell migration

**Figure 4:**
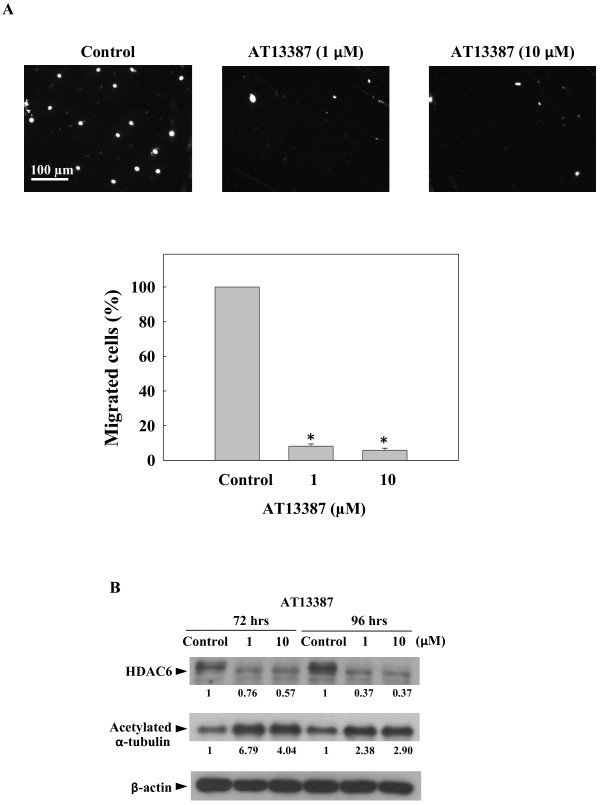
**Inhibitory effect of AT13387 on C666-1 cell migration. (A)** Trans-well migration assay. The C666-1 cells treated with AT13387 for 72 hrs were harvested and viable cells were seeded on the upper chamber of transwell for migration assay. Upper panel: images of cells migrated through the membrane of migration chamber stained with DAPI. Scale bar = 100 μm. Lower panel: quantification of migrated cells. At least 100 cells were counted from different microscopic fields. Results were expressed as the mean ± S.D. of three separate trials; *p < 0.05. **(B)** Western-blotting analysis of C666-1 treated with AT13387 for 72 hrs and 96 hrs showed decreased expression of cell migration regulator HDAC6 and increased acetylated form of the α-tubulin. Value represented the arbitrary unit of band intensity after normalization with β-actin.

Tumor cell metastasis is one of the current problems in the treatment of NPC, the migration capability of AT13387-treated C666-1 cells was then evaluated using a transwell migration assay. The C666-1 cells pre-treated with AT13387 for 72 hours were harvested and seeded on the upper chamber of transwell for migration assay. Cells migrated through the membrane of migration chamber were stained with DAPI and at least 100 cells per treatment were counted from different microscopic fields. Figure 4A showed the migration capability of AT13387-treated C666-1 cells was significantly reduced (*p < 0.05). At the concentration of 1 μM and 10 μM, the percentage of migrated cells was reduced to 8% and 5%, respectively, compared to the untreated control. Since the assembly (acetylation of α-tubulin) and disassembly (deacetylation of α-tubulin) of microtubule is important in cell migration. Next, we determined the expression of a known microtubule-associated deacetylase, histone deacetylase 6 (HDAC6), and the acetylation status of α-tubulin in AT13387-treated C666-1 cells. HDAC6 is a cell migration regulator and it is also client protein of Hsp90 [28-30]. Results in Figure 4B showed that the expression of HDAC6 was greatly diminished in cells after AT13387 treatment. The effect was accompanied with an increased in the expression of acetylated form of the α-tubulin. This finding suggested that the migration inhibitory activity of AT13387 might be due to the disruption of the microtubule dynamic through the reduction of the expression of HDAC6.

### AT13387 inhibits the tumor spheres formation and growth, accompanied by reduction of CD44 and SOX2 expression

**Figure 5:**
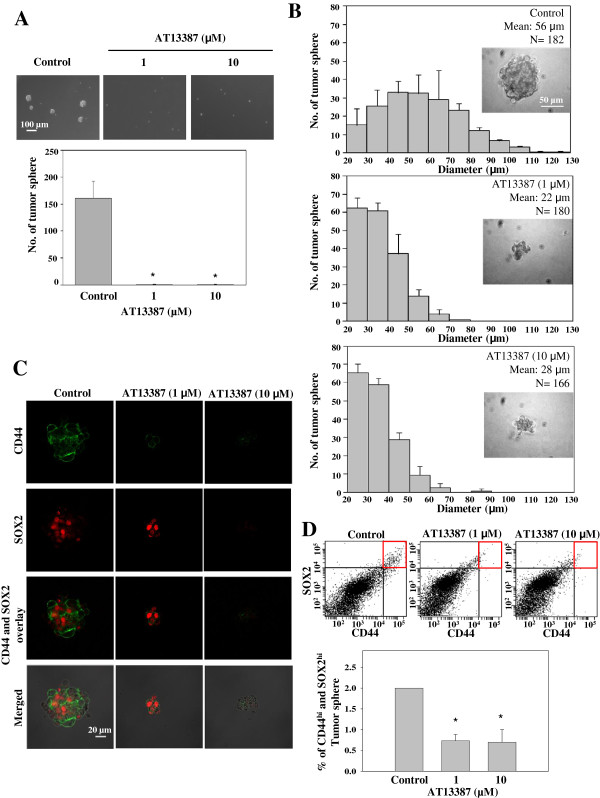
**Effect of AT13387 on C666-1 tumor sphere. (A)** AT13387 was added on day 0 of C666-1 tumor sphere formation. Upper panel: image of tumor spheres formed from C666-1 single cell culture with and without AT13387 treatment. AT13387 significantly inhibited tumor sphere formation. Scale bar = 100 μm. Lower panel: Total number of tumor spheres per culture. Tumor spheres formed with diameter reaching 20 μm were counted. Results were expressed as the mean ± S.D. of three experiments; *p < 0.05. **(B)** Size profile of C666-1 tumor spheres. AT13387 was added to the tumor sphere culture after tumor spheres were formed for 7 days. The diameters of tumor spheres were measured after AT13387 treatment for 7 more days and presented as the size profile of tumor sphere. The mean diameters of tumor spheres with AT13387 treatment were significantly smaller than the untreated control (*p* < 0.05), showing the inhibitory effect of AT13387 on the growth of C666-1 tumor sphere. Scale bar = 50 μm. Results were expressed as the mean ± S.D. of three experiments. **(C)** Confocal image showing pseudocolor green for CD44 and red for SOX2. Loss of CD44 was observed in 1 μM AT13387-treated C666-1 tumor sphere and loss of both CD44 and SOX2 were observed in 10 μM AT13387-treated C666-1 tumor sphere. Scale bar = 20 μm. **(D)** FACS analysis showing the decrease of CD44^hi^ and SOX2^hi^ population in AT13387-treated C666-1 tumor sphere. Results were expressed as the mean ± S.D. of three experiments; *p < 0.05.

3-D tumor sphere formation assay is frequently used as an *in vitro* assay to evaluate the clonogenicity of tumor cells. This method is also frequently used to measure the growth of putative cancer stem cells (CSCs) under the serum-free and an ultra-low attachment conditions [31-33]. In a recent EBV-associated NPC cancer stem-like cells study, the CSC population in C666-1 tumor spheres were found to have upregulation of multiple stem cell markers and high tumor initiating ability in nude mice. Both CD44 and SOX2 CSC-like markers were overexpressed in the C666-1 tumor sphere and the isolated CD44^+^ NPC cells were found to be more resistant to chemotherapeutic agent [34]. In the present study we further examined the inhibitory effect of AT13387 on C666-1 tumor spheres. Total number of tumor spheres having diameter >20 μm in each culture were counted and compared. Figure 5A showed AT13387 completely inhibited the formation of C666-1 tumor spheres. The C666-1 cells treated with AT13387 remained as single cell while tumor spheres were formed in the untreated culture in 7 days. Next, we further studied the inhibitory effect of AT13387 on the growth of established tumor spheres. AT13387 was added on day-7 after the initiation of tumor sphere formation assay. Results in Figure 5B showed the representative images and size profiles of untreated tumor spheres and tumor spheres after AT13387 treatment for another 7 days. The mean diameter of control tumor spheres was 56 μm while the mean diameter of 1 μM and 10 μM AT13387-treated tumor spheres were 22 μm and 28 μm, respectively. The AT13387-treated tumor spheres were significantly smaller than the untreated control (*p* < 0.05), showing the inhibitory effect of AT13387 on the growth of C666-1 tumor sphere. We then studied the effect of AT13387 on CD44 and SOX2 in C666-1 tumor spheres. Figure 5C showed the confocal image of CD44 (green)-and SOX2 (red)-stained tumor spheres. Highly reduced expression of CD44 was observed in 1 μM AT13387-treated tumor sphere and loss of both CD44 and SOX2 were observed in 10 μM AT13387-treated tumor sphere. We further quantified the reduction of CD44 and SOX2 expression by Fluorescence-activated Cell Sorting (FACS) analysis. In Figure 5D, the upper panel showed the dot-plot of CD44 and SOX2 stained cells. The CD44^hi^ and SOX2^hi^ populations were indicated by red squares and quantified in a bar chart presented in the lower panel. Result showed there was a 3-fold reduction of CD44^hi^ and SOX2^hi^ populations in 1 μM and 10 μM AT13387-treated C666-1 tumor spheres compared with the untreated control tumor spheres (*p < 0.05). Both the immunofluorescence staining and FACS analysis showed AT13387 significantly reduced the CD44 and SOX2 expression in C666-1 tumor spheres.

### AT13387 suppressed NPC tumor formation in nude mouse tumorigenicity assay

**Figure 6:**
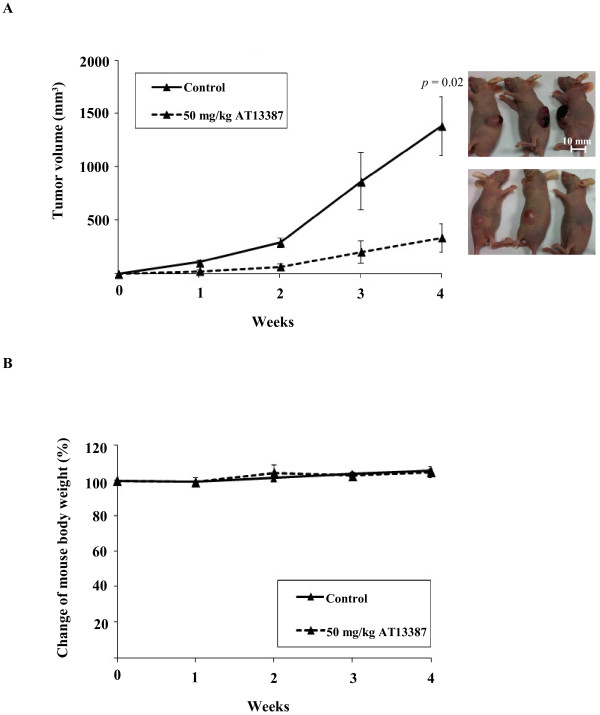
**AT13387 suppressed tumor formation in nude mouse tumorigenicity assay. (A)** The tumor volume of AT13387-treated mice and vehicle control mice (n = 3) were measured weekly. The solid line represented the average tumor volume of vehicle control group, which reached 1300 mm^3^ in week 4. The dashed line represents the average tumor volume of AT13387-treated mice which showed significant suppression of tumor formation when compared to vehicle control. **(B)** The mouse body weight of AT13387-treated mice and control mice during the experiment. The result showed no significant difference for mouse body weight after AT13387 drug treatment group when compared to control group.

The antitumor effect of AT13387 *in vivo* was studied using the nude mouse tumorigenicity assay. The nude mice were subcutaneously injected with 1×10^7^ C666-1 cells. After cell inoculation, the mice were randomly divided into two groups to receive either 50 mg/kg AT13387 treatment or vehicle control through i.p. injection twice a week for a total of 4 weeks. The tumor volume and body weight of the mice were measured weekly. Figure 6A showed that the average tumor volume of the vehicle control group which reached 800 mm^3^ by week 3 and continued to grow and exceeded 1300 mm^3^ by week 4. For the AT13387 treatment group, the average tumor volume reached 200 mm^3^ at week 3, but did not exceed 400 mm^3^ until week 4. AT13387 significantly suppressed tumor formation in nude mice (p = 0.02), with no adverse effect on mice body weight (Figure 6B) and no apparent harmful effects, when compared to the control mice receiving vehicle alone.

## Discussion

Cancer is a complex disease, with multiple aberrantly overexpressed oncogenic proteins involving activation of multiple signaling pathways. The stability of most of these oncoproteins depends heavily on the chaperon function of Hsp90. For this reason, the molecular chaperone Hsp90 is an attractive therapeutic target in cancer therapy. In the present study, we demonstrated both the *in vitro* and *in vivo* antitumor effects of a novel Hsp90 inhibitor, AT13387, on C666-1 EBV-positive NPC cells. First of all, AT13387 was found to inhibit cell growth and induce cellular senescence in the C666-1 EBV-positive NPC cells. Inhibition of cell growth and induction of cellular senescence, instead of induction of cell death through Hsp90 inhibition has also been reported in small cell lung cancer as a mode of cancer cell response to Hsp90 inhibitor [35]. Cellular senescence is a permanent and irreversible process in the induction of cell growth arrest without massive cell death [20,21]. The induction of cellular senescence has recently been proposed as a novel approach to improve cancer therapy with less severe side effects than cytotoxic therapies and high dose radiation [36,37].

In the present study, AT13387 was found to downregulate several cell growth and cellular senescence associated Hsp90 client oncoproteins, including CKD4, AKT and EGFR. Also, we reported the correlation between restoration of p27 protein expression and the downregulation of S-phase kinase associated protein 2 (Skp2). Skp2 is the F-box protein responsible for substrate recognition in the Skp1-Cullin1-F-box (SCF) E3 ubiquitin ligase and specifically targeting the tumor suppressive proteins such as p27 for ubiquitination and proteasomal degradation [24]. The role of the Skp2 in the regulation of cellular senescence has recently been reported [38] and reviewed [39]. In the present study, we found that AT13387 induced senescence in C666-1 cells and the effect was correlated with the reduction of the Skp2 and the increased expression of p27. The stability of Skp2 has been reported to be dependent on the phosphorylation by AKT [40]. We further demonstrated that the loss of Skp2 was correlated with the reduced expression of the Hsp90 client proteins AKT in the treated C666-1 cells. These findings suggested that AT13387 inhibit cell growth and induce cellular senescence in C666-1 by downregulating cell growth and cellular senescence associated Hsp90 client proteins and also restored the tumor suppressive protein p27 by downregulating Skp2 through downregulation of Hsp90 client protein AKT and p-AKT.

The downregulation of Skp2 by AT13387 showed an important clinical relevance in the treatment of NPC which is worthy to discuss. Recent studies on the clinical samples from Taiwan and South China showed that Skp2 was overexpressed in 80% NPC tumor and the expression was correlated with poor prognosis [25,26]. The overexpression of Skp2 in NPC clinical samples may explain the commonly loss of p27 in NPC tissues [41,42]. The oncogenic role of Skp2 in NPC pathogenesis has been studied in NPC cells transfected with Skp2 of showing higher colony forming ability and the side population of NPC cells showed higher level of Skp2 [25,26]. However, up until now, no pharmacological Skp2 inhibitor has yet been available for clinical use. In our study, we demonstrated Skp2 can be downregulated by AT13387 in C666-1. This observation suggested that NPC patients with a high Skp2 expression might benefit from AT13387 for personalized therapy.

As mentioned above, AT13387 can target on multiple oncoproteins simultaneously. We studied the depletion of a very important NPC oncoprotein EGFR in AT13387-treated C666-1. EGFR has been reported to be overexpressed in 85% of NPC tissues and the expression is associated with poor prognosis [43]. EGFR is the receptor tyrosine kinase of the natural ligand EGF and TGF. Activation of EGFR was associated with proliferation, migration, and drug resistance, which play an important role in NPC pathogenesis. In recent years, EGFR has been proposed as a new therapeutic target for NPC. EGFR inhibitors such as cetuximab and gefitinib, which are the monoclonal antibody and the small molecule against EGFR, respectively, are currently under NPC clinical evaluations [44]. However, targeting a single oncoprotein is unlikely to be effective enough to eliminate the disease, as the tumor cells may switch from utilization of one signaling pathway to another signaling pathway for growth [45]. Despite the promising effect of EGFR inhibitors in the preclinical and clinical studies, not all the patients respond and benefit from the treatment in clinical studies [46,47]. In one third of gefitinib non-responsive NPC patients, AKT was found to be overexpressed [48]. The activation of AKT pathway in gefitinib-resistant cells may take over the EGFR pathway and therefore maintain the tumorigenicity and escape from the EGFR targeted therapy. In the present study, we observed the simultaneous downregulation of EGFR, EGFR downstream signaling molecules p-STAT3, AKT and p-AKT. Hence, targeting multiple oncoproteins using AT13387 alone or in combination with specific antitumor agents may serve as a potential solution to overcome the development of drug resistance in NPC targeted therapy.

One of the current problems in the treatment of NPC is the development of distant metastasis and tumor recurrence. HDAC6, also a client protein of Hsp90 [29], is a key modulator involved in the regulation of cell migration through the deacetylation of tubulins in the cytoplasm [28,30,49]. Overexpression of HDAC6 is frequently correlated with the tumor development, and hence HDAC6 is considered to be a target for cancer therapy [50]. However, the role of HDAC6 in NPC has not been demonstrated. In the present study, we found that the expression of HDAC6 was downregulated by AT13387. The effect was correlated with the increase in the acetylation of α-tubulin and the decrease in the tumor cell migration. This finding indicates that AT13387 may reduce metastasis through the disruption of microtubules dynamics.

In addition to the mechanistic study, two biological end-point assays, namely the *in vitro* 3D tumor sphere formation assay and the *in vivo* NPC xenograft, were used to evaluate the efficacy of AT13387 for NPC. The tumor sphere assay is frequently used to measure the *in vitro* self-renewal capability of cancer stem cells and to assess the effectiveness of the drug on the cells in the presence of growth factors [31-33]. Our results clearly showed that AT13387 not only reduced the *in vivo* tumor formation, but also reduced the formation and growth of NPC tumor spheres accompanied by reduced expression of cancer stem-like cells markers CD44 and SOX2. Lo KW and co-workers have recently demonstrated that CD44 and SOX2 expression are enriched in C666-1 tumor sphere forming cells which may serve as the potential candidate stem cell markers for the NPC C666-1 cells [34]. CD44 is a well known cell surface marker involved in the signal transduction of multiple oncogenic pathways [51-53]. SOX2 is a well known master transcription factor of stem cells [54]. Decreased expression of CD44 and SOX2 might reduce the oncogenic potential of the tumor cells. The result revealed the potential of AT13387 on targeting the CD44-and SOX2-overexpressing NPC subpopulation. Taken together, results from the present study suggest that targeting on multiple oncogenic pathways by AT13387 is a novel approach in the treatment of NPC. Further development will focus on the evaluation of using AT13387 as a single agent or in combination with other current therapies in the treatment of NPC.

## Conclusion

Our study demonstrated the *in vitro* and *in vivo* antitumor effect of a novel Hsp90 inhibitor, AT13387, on the EBV-positive NPC cell line C666-1. AT13387 inhibited cell growth, cell migration, tumor sphere formation and induced cellular senescence in C666-1. The ability of AT13387 to target multiple NPC oncoproteins, make it a potent antitumor agent in treatment of NPC. Together with the tumor suppressive effect of AT13387 in nude mice tumorigenicity assay, this study provided preclinical evidence of using AT13387 as a new therapeutic agent in treatment of NPC.

## Methods

### Chemical and antibodies

AT13387 was synthesized and provided by Astex Pharmaceuticals Inc [13]. AKT inhibitor SH-6 was purchased from Calbiochem, San Diego, CA. Primary antibodies for Western blotting analysis include Caspase-3, BAX, Bcl-2, Bcl-xl, CDK2, CDK4, p16, p21, p27, Rb, STAT3, p-STAT3 (Tyr705), AKT, p-AKT (Ser473), Skp2 (Cell Signaling, Danvers, MA); EGFR, Hsp90, and HDAC6 (Santa Cruz Biotechnology, Santa Cruz, CA); and acetylated α-tubulin and β-tubulin (Sigma-Aldrich, St. Louis, MO). Antibodies for immunofluorescence staining were Alexa Fluor® 488 conjugated CD44 and Alex Fluor® 647 conjugated SOX2 (Cell Signaling, Danvers, MA).

### Cell line and cell culture

C666-1, an EBV-positive NPC cell line still carrying the native EBV genomes [3], were cultured in RPMI-1640 medium (Invitrogen, Carlsbad, CA) supplemented with 10% fetal bovine serum (FBS) (Invitrogen, Carlsbad, CA) and 1% penicillin and streptomycin (P/S) (Invitrogen, Carlsbad, CA). Cells were cultured at 37°C with 5% CO_2_ in humidified incubator.

### MTT cell viability assay

Viable cells treated with various dose of AT13387 were measured by 3-(4,5-dimethylthiazol-2-yl)-2,5-diphenyltetrazolium bromide (MTT) assay as previously described [55]. Briefly, C666-1 (3×10^4^) were seeded in 96-well microplates and treated with serial diluted AT13387 (0.001 μM-10 μM) for 48 hours. MTT solution (Sigma-Aldrich, St. Louis, MO) (0.25 mg⁄ml) was added to cells and incubated for 3 hours in 37°C. The optical densities (OD) were measured at absorbance 550 nm with reference to absorbance 690 nm. The OD is directly proportional to the number of living cells and the percentage of viable cells compared to control wells was calculated.

### Cell growth assay

The kinetic effect of AT13387 on proliferation of C666-1 was studied using a cell growth assay. C666-1 cells (3×10^5^) were seeded onto 35 mm culture dishes. The cells were then treated with AT13387 (1 μM and 10 μM) for 2 to 7 days. The total number of viable cells determined by trypan blue staining was counted on day 2, 4, and 7 after AT13387 treatment.

### DNA content analysis

DNA content analysis was performed using propidium iodide (PI) staining and flow cytometry analysis as previously described [56]. Briefly, C666-1 (3×10^5^) were seeded in 6-well plates and treated for 48 hours with 1 μM ATT13387 (the minimum concentration that had shown maximum cell growth inhibition in MTT assay). Both adherent cells and floating cells were collected for analysis. The cells were fixed in 70% cold ethanol, stained with 1 mg/ml propidium iodide (PI) and analyzed by FACSCalibur flow cytometer (Becton Dickinson, Franklin Lakes, NJ). Fluorescence profiles represent the DNA content of the PI stained cells.

### Nucleus and SAHF staining with DAPI

DAPI nucleus staining was used to identify the apoptotic cells with chromatin condensation and fragmentation and/or senescence cells with senescence-associated heterochromatic foci (SAHFs) formation as previously described [22,57]. For the apoptotic nucleus staining, 3×10^5^ cells were seeded in 6-well plates and treated with 1 μM AT13387 for 48 hours. For the SAHF staining, 3×10^5^ cells were seeded in 6-well plates and treated with 1 μM and 10 μM AT13387 for 96 hours. Both adherent cells and floating cells were collected onto slides by cytospin. The cells were fixed with 2% paraformaldehyde and permeablized with 0.2% Triton-X. The cells were then stained with DAPI (1 μg/ml) and the nuclear images were captured under a fluorescence microscope (ECLIPSE Ti, Nikon) equipped with camera. At least 200 cells were counted from different microscopic fields.

### Senescence-associated β-Galactosidase cell staining

Senescence-associated β-galactosidase (SA-β-gal) activation was detected by cytochemical staining with the X-Gal according to the protocol of the Cell Signaling Senescence β-Galactosidase Staining Kit #9860. Briefly, C666-1 (8×10^4^) cells were seeded onto wells of a 24-well plate and the cells were treated with 1 μM and 10 μM of AT13387 for 72 hours. Both adherent cells and floating cells were collected and stained with X-gal (pH6) overnight in the dark. The senescent cells were stained with blue color. Cells images were captured under microscope with a camera.

### Western blotting analysis

Western blotting analysis was performed as previously described [56]. In brief, C666-1 cells (5×10^5^) were seeded onto the 6-well plate and the cells were treated with 1 μM and 10 μM of AT13387 for 48 hours, 72 hours and 96 hours. Both adherent cells and floating cells were collected and lysed with ice-cold lysis buffer (250 mM Tris–HCl, pH 8; 1% NP-40 and 150 mM NaCl containing 1% phosphatase inhibitors cocktail (Calbiochem, San Diego, CA) and 0.25% protease inhibitors cocktail (Sigma, St. Louis, MO). Samples were resolved on SDS–polyacrylamide gel and transferred to PVDF membrane (Millipore, Billerica, MA). The membrane was blocked with 5% non-fat milk, incubated with primary antibodies (1:1000) followed by corresponding secondary antibodies (1:4000). A Western blotting substrate (Labfrontier Co. Ltd., Bio Division) was added and chemiluminescence signal was detected on the X-ray film. β-actin primary antibody (1:4000) was probed and served as an internal control.

### siRNA knockdown

Knockdown with siRNA and transfection were performed according to the manufacturer’s instruction. In brief, C666-1 cells (5×10^5^cells/well) were seeded onto fibronectin-coated 35-mm dishes for 24 hours. Cells were transfected with 5 nM of si-Skp2 RNA (a pool of 4 siRNA: GGAUGUGACUGGUCGGUUG; GGUAUCGCCUAGCGUCUGA; UCGGUGCUAUGAUAUAAUA; UGUCAAUACUCUCGCAAAA), or si-Control RNA (UGGUUUACAUGUCGACUAA) (Dharmacon, Lafayette, CO) using Lipofectamine Reagent 2000 (Invitrogen, Carlsbad, CA). After 6 hours of transfection, the medium was then replaced by fresh medium. Cells were harvested for western-blotting analysis after 72 hours of transfection.

### Migration assay

The migration capability of AT13387-treated C666-1 cells was analyzed using the transwell migration assay. C666-1 cells (5×10^5^) were seeded on the 6-well plate and treated with 1 μM and 10 μM AT13387 for 72 hours. Cells were then harvested and 2×10^5^ viable cells were seeded on the upper chamber of the transwell. After 24 hours of incubation, the cells that had migrated through the membrane were fixed in 2% paraformaldehyde, permeablized with 0.2% Triton-X, and stained with 1 μg/ml DAPI. The stained cell images were captured under fluorescence microscopy (ECLIPSE Ti, Nikon). At least 100 cells were counted from different microscopic fields.

### Tumor sphere formation assay

Tumor sphere formation assay was performed as previously described [32]. C666-1 cells were dissociated into single cells and seeded in low cell density (2×10^3^) on a 24-well ultra-low attachment plate (Corning, Acton, MA), and cultured with serum-free DMEM/F-12 (Invitrogen, Carlsbad, CA), 20 ng/ml EGF (Sigma, St. Louis, MO), 20 ng/ml bFGF (Cell Signaling, Danvers, MA), and 20 ng/ml insulin (Cell Signaling, Danvers, MA). The cultures were fed with fresh serum-free DMEM/F12 supplemented with growth factors every other day. For studying the effect of AT13387 on the tumor sphere forming ability, AT13387 was added to the culture on the same day of seeding the dissociated C666-1 single cells. After 7-days of incubation, the images of cells were captured under an inverted microscope equipped with camera. Tumor spheres having diameter >20 μm were counted using Image J software. Total numbers of tumor spheres formed in AT13387-treated and-untreated cultures were compared. In order to study the effect of AT13387 on the growth of established tumor spheres, tumor spheres were first allowed to grow for 7 days, followed by incubation with AT13387 for further 7 days. Then the images of AT13387-treated and–untreated tumor spheres were captured under an inverted microscope equipped with camera. Tumor spheres with diameter >20 μm were measured and counted using Image J software. Data from each treatment were presented as size distribution profile with mean diameter.

### Immunofluorescence staining and FACS analysis of spheroid cells

Tumor spheres for CD44 and SOX2 immunofluorescence staining and FACS analysis were established as described above. Briefly, C666-1 cells were incubated in serum-free DMEM/F12 supplemented with growth factors for 7 days to allow tumor sphere formation. Then, AT13387 was added to the tumor spheres culture and incubated in serum-free DMEM/F12 supplemented with growth factors for another 7 days. For CD44 and SOX2 immunofluorescence staining, the tumor spheres were carefully collected and fixed with 2% paraformaldehyde and permeablized with 0.2% Triton-X. Tumor spheres were then incubated with Alexa Fluor® 488 conjugated CD44 and Alex Fluor® 647 conjugated SOX2 antibodies in the dark. The immunofluorescence signals were visualized and imaged using an Olympus Fluoview 1000 confocal scanning laser microscope. For FACS analysis, tumor spheres were collected and the spheroid cells were resuspended in PBS and fixed in 1.6% paraformaldehyde. Then the cells were pelleted and resuspended in ice-cold methanol. The cells were washed twice in incubation buffer [0.5% bovine serum albumin (BSA) in PBS], and stained with Alexa Fluor® 488 conjugated CD44 and Alex Fluor® 647 conjugated SOX2 antibodies in the dark. Respective mouse or rabbit IgG isotypic controls were included as negative controls. For each sample, 10,000 cells were acquired and analyzed by FACSCalibur flow cytometer (Becton Dickinson, Franklin Lakes, NJ).

### Nude mice tumorigenicity assay

Nude mice were supplied and housed by Laboratory Animal Unit of the University of Hong Kong. Experiments were conducted under license from the Hong Kong Department of Health and approved by Committee on the Use of Live Animals in Teaching and Research (CULTAR) at the University of Hong Kong. AT13387 drug formulation used in a previous publication [14] was used in the nude mice tumorigenicity assay. In brief, 1×10^7^ C666-1 cells were subcutaneously (s.c.) injected into the flank of 8-10 week old female athymic BALB/c nu/nu mice. Immediately after cell inoculation, the mice were randomly divided into two groups (three mice per group) for either treatment with AT13387 or vehicle. For the drug treatment group, AT13387 formulated in 17.5% hydroxy-propyl-β-cyclodextrin in sterile water was administrated at 50 mg/kg by intraperitoneal (i.p.) injection at a dose volume of 10 ml/kg twice per week (on Days 2 and 5 of each week). For the control group, the drug vehicle alone was given through i.p. injection. The tumor volume in mm^3^ (length × width × height) and the mice body weight were measured weekly until tumor volume reached 1000 mm^3^.

### Statistical analysis

All results were representative results from at least two independent experiments. Each data points with error bars were the arithmetic mean ± SE of three replicates (n = 3). The *p*-values were calculated using Student’s *t*-test, *p*-value < 0.05 was considered as statistically significant.

## Competing interests

The authors declare that they have no competing interests.

## Authors’ contributions

Designed the experiments: KCC and NKM; Performed the experiments and analyzed data: KCC, CMT, PSC, and MCL; Wrote the manuscript: KCC; Edited the manuscript: KCC, KWL, JEC, TS, AWML, WTN, GSWT, RNSW, MLL and NKM. All authors read and approved the final manuscript.
